# Photodynamic Therapy Activated by Intense Pulsed Light in the Treatment of Nonmelanoma Skin Cancer

**DOI:** 10.3390/biomedicines6010018

**Published:** 2018-02-07

**Authors:** Domenico Piccolo, Dimitra Kostaki

**Affiliations:** 1 Italian Association Outpatient Dermatologists, 00177 Rome, Italy; 2 Skin Center, Dermo-Aesthetic Lasers Centers, 67051 Avezzano, Italy; kostakidimi@hotmail.com

**Keywords:** photodynamic therapy, nonmelanoma skin cancer, MAL, intense pulsed light device

## Abstract

Photodynamic therapy (PDT) with topical 5-aminolevulinic acid (ALA) or methyl aminolevulinate (MAL) has proven to be a highly effective conservative method for the treatment of actinic keratosis (AK), Bowen’s disease (BD), and superficial basal cell carcinoma (sBCC). PDT is traditionally performed in association with broad-spectrum continuous-wave light sources, such as red or blue light. Recently, intense pulsed light (IPL) devices have been investigated as an alternative light source for PDT in the treatment of nonmelanoma skin cancers (NMSC). We herein report our observational findings in a cohort of patients with a diagnosis of AK, sBCC, and BD that is treated with MAL-PDT using IPL, as well as we review published data on the use of IPL-PDT in NMSC.

## 1. Introduction

Nonmelanoma skin cancer (NMSC) is the most common malignancy among the Caucasian population [1]. Over the last decades, the worldwide incidence is estimated to rise by 3–10% [2], presenting an increasing demand on healthcare resources.

Surgery is the traditional “gold-standard” treatment since it provides high rates of disease control with clear identification of tumour margins [3]. NMSC are slow-growing skin tumours that are characterized by low risk of metastatic potential and favourable life prognosis; therefore, conservative therapies should be considered based on lesion’s characteristics (location and size), as well as patient’s specific factors (age, comorbidity, medications, immunosuppression).

Photodynamic therapy (PDT) with topical 5-aminolevulinic acid (ALA) or methyl aminolevulinate (MAL) has proven to be a highly effective conservative method for the treatment of actinic keratosis (AK), Bowen’s disease (BD), and superficial basal cell carcinoma (sBCC), with the benefit of excellent cosmetic results and the potential for field treatment [4].

Originally developed to be used with broad-spectrum continuous-wave light sources such as red or blue light for the treatment of superficial cutaneous malignancies, PDT has more recently been used in combination with other light sources, including light-emitting diodes (LEDs), lasers, and incoherent light sources, with all giving adequate complete remission rates [5].

Intense pulsed light (IPL) is a source of incoherent light that was developed in the early 1990s that targets several chromophores, such as melanin, vascular structures, and collagen, thus improving photoaged skin.

The rationale for using the IPL as a light source for PDT is based on the absorption spectrum of the photosensitizer and IPL’s features. Protoporphyrin IX (PpIX) has absorption peaks at 505, 540, 580, and 630 nm. IPL is a source of incoherent light with emission spectrum ranging of 500–1200 nm. By using different cut-off filters in the handpiece, the delivered wavelengths can be varied, underlying IPL’s versatility. Thus, the adaptation to the absorption spectrum of PpIX allows for the use of IPL for PDT [6].

A major advantage of IPL-PDT as compared with conventional PDT is less time expenditure and less painful effects of heat delivery due to shorter intense exposure times [7]. IPL can also penetrate much deeper and trigger greater photodynamic reactions than other types of light sources.

Although the use of IPL for ALA activation in the treatment of AK has been addressed in several studies, particularly as part of photorejuvenation process [8,9,10,11,12], there is a scarcity of literature describing the use of IPL for MAL activation in the treatment of AK, as well as sBCC and BD. MAL is reported to have increased lipophilicity and deeper skin penetration when compared with ALA. However, there is no statistically significant difference in efficacy between ALA and MAL in the treatment of AK [13].

We herein report our observational findings in a cohort of patients with a diagnosis of AK, sBCC, and BD treated with MAL-PDT using IPL as light source as well as we review published data on the use of IPL-PDT in NMSC.

## 2. Patients and Methods

### 2.1. Patients

Twenty-five patients with AK, sBCC, and BD were enrolled. All of the patients were poor surgical candidates as their lesions were large, multiple, or in cosmetically sensitive areas. Diagnosis was established clinically and dermoscopically by two experienced dermatologists while biopsy specimens were taken in case of diagnostic doubt. Written informed consent regarding the potential benefits and risks of the procedure was obtained from each patient. Since MAL cream licence does not specify a particular light source for PDT, and these observations were carried out during our routine clinical practice, ethical approval was not sought.

### 2.2. Treatment Protocol

Immediately prior to PDT, all of the hyperkeratotic lesions were treated with CO_2_ laser to increase cream and light penetration. MAL (Metvix^®^, Galderma Italia S.p.A, Agrate Brianza, Italy) was applied to lesion as a 1-mm thick layer, including 5 mm of the surrounding normal tissue, while in cases of multiple lesions it was applied to entire anatomic area. An occlusive nonabsorbent wrap and aluminium foil were placed over the MAL to increase penetration and eliminate light exposure, respectively.

After an occlusion time of 3 h, MAL was removed. Before irradiation, the fluorescence of the lesion that was treated with MAL was detected by a Wood’s lamp photofluorescence. Subsequently, a thin layer of chilled gel was applied and irradiation was performed. All patients and investigators wore protective goggles during treatment.

MAL was activated by an IPL device (Photosilk plus, DEKA M.E.L.A. S.r.l, Calenzano, Italy) set to the following parameters: cut-off wavelength, 550 nm; fluence, 18 J/cm^2^; triple pulse mode, 3.3, 3.9, and 4.6 ms in duration; interpulse delay, 100 ms with epidermal cooling already provided by the IPL handpiece. Patients with AK received three passages of irradiation in a single treatment session, while patients with sBCC and BD underwent four passages of irradiation in two treatment sessions at two-week interval. No analgesia or local anaesthesia was given. Post treatment care was limited to a topical antibacterial ointment and a sunscreen. Patients were strictly advised to avoid sunlight and to use sunscreen for the following 6 weeks.

Clinical (both two-dimensional (2D) and three-dimensional (3D)) and dermoscopic images were captured in all of the cases before each treatment and six months following the last treatment. A special lens for dermoscopy (DermLite Foto; 3Gen LLC, San Juan Capistrano, CA, USA) connected to a digital camera (Canon PowerShot A360, Tokyo, Japan) was used for dermoscopic images, while a digital camera (Canon EOS 350D) and the 3D LifeViz^®^ Micro system was used for two- and three-dimensional imaging of the lesions, respectively.

### 2.3. Outcome Assessment

Efficacy was evaluated by two dermatologists through inspection, palpation and dermoscopic evaluation 6 months after treatment and classified as either in complete response (complete disappearance of tumour) or as partial response.

The cosmetic outcomes of patients were assessed and graded as excellent (absence of any sign of treatment), good (visible fibrosis, atrophy, or change in pigmentation), poor (moderate visible fibrosis, atrophy, or change in pigmentation), or fair (marked visible fibrosis, atrophy, or change in pigmentation).

Pain during treatment was recorded on a visual analogue scale (VAS) of 0 to 10 with 0 indicating no pain and 10 insufferable pain.

## 3. Results

The study included 25 patients, 15 men and 10 women (age range: 54–93 years, mean: 71.3 years), with a total of 29 lesions, including AK (20), sBCC (5), and BD (4).

**Figure 1:**
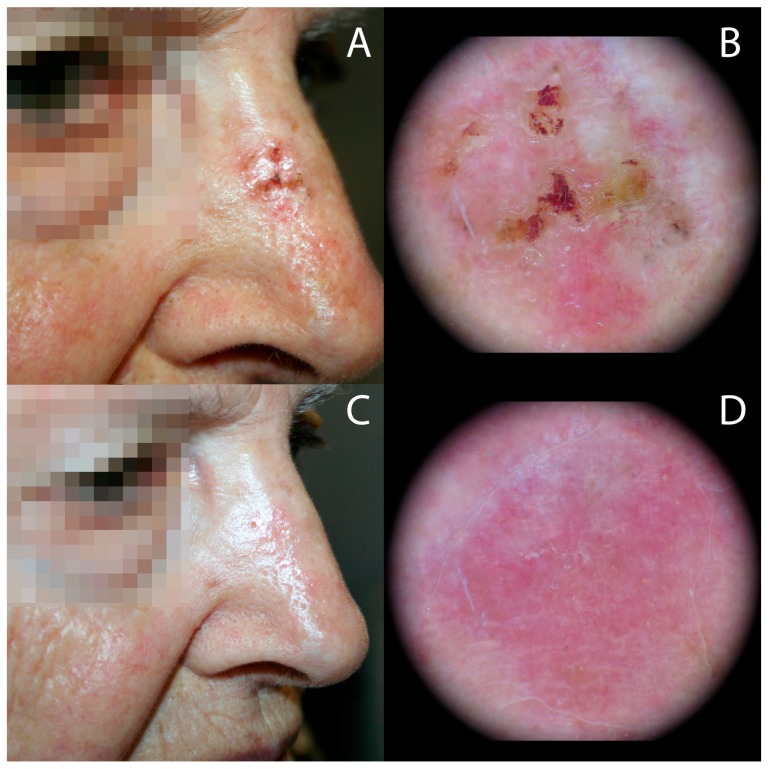
Superficial basal cell carcinoma (sBCC) at the right side of the nose treated with methyl aminolevulinate (MAL)-photodynamic therapy (PDT) using intense pulsed light (IPL) as light source. Clinical and dermoscopic images showing: (**A**,**B**) short fine telangiectasias, multiple small erosions located in a red structureless background and scales before treatment and (**C**,**D**) complete clinical and dermoscopic respone at six months follow-up. Cosmetic outcome was rated as excellent.

**Figure 2:**
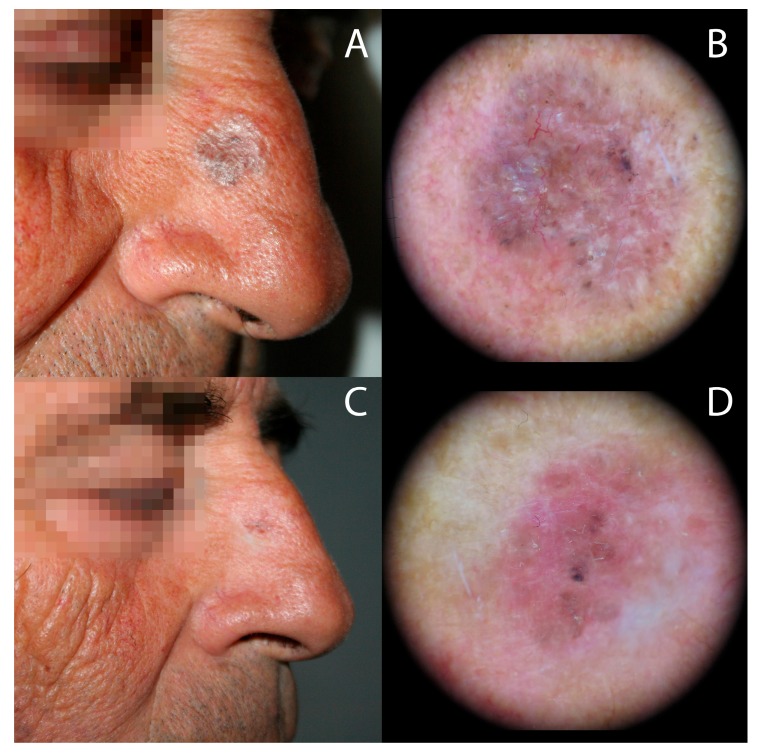
Superficial pigmented BCC at the right side of the nose treated with MAL-PDT using IPL as light source. Clinical and dermoscopic images showing: (**A**,**B**) arborizing telangectasias, large blue/gray ovoid nests, multiple blue/gray globules before treatment and (**C**,**D**) partial clinical and dermoscopic response, defined by a decrease of BCC-specific dermoscopic features with persistence of few arborizing telangectasias and blu/gray globules, at six months follow-up.

**Figure 3:**
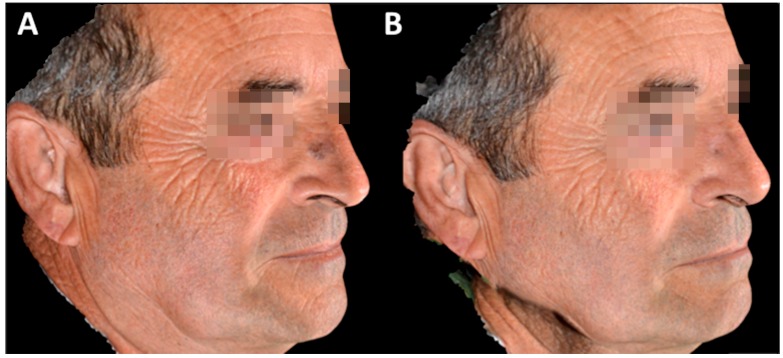
Three-dimensional (3D) images of partial clinical response: (**A**) before and (**B**) at 6 months following MAL-PDT using IPL as light source.

Table 1 demonstrates the results at six months follow-up. For AK, clinical and dermoscopic response was complete in 18 of the 20 lesions (90%), and partial response in the rest (10%). Complete response was found in 4/5 sBCC (80%) (Figure 1) and in 4/4 BD (100%), while partial response was observed in 1/5 sBCC (20%) (Figure 2 and Figure 3). In summary, 26 of 29 lesions (89.6%) showed clinical and dermoscopic resolution after one or two IPL-PDT, according to the treatment protocol.

All of the patients experienced mild pain during lesion irradiation as well as slight warmth after treatment for less than 20 s; however, in no case was treatment interruption required. After each session, all of the patients experienced transient mild edema and erythema, followed by appearance of crusting with healing within 1 week.

**Figure 4:**
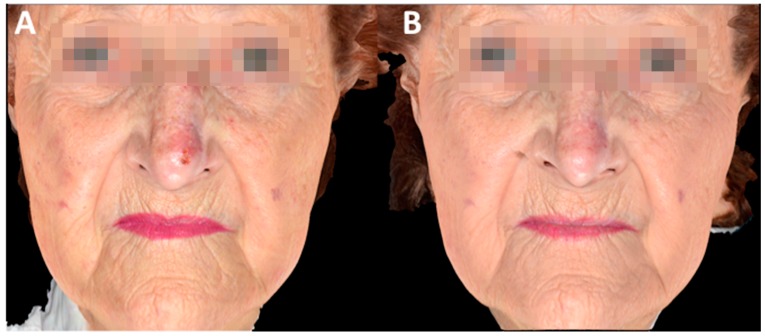
3D images of complete clinical response of AK at the nose treated with MAL-PDT using IPL as light source: (**A**) before treatment and (**B**) at six months follow-up. Cosmetic outcome was rated as excellent.

At six months follow-up the cosmetic outcome was excellent in 29 of 29 (100%), of which two cases are demonstrated in Figure 1 and Figure 4.

## 4. Discussion

Our observations confirm that IPL might be a potential alternative light source for PDT in the treatment of NMSC. The six-month complete response rate of 89.6% is consistent with published short-term treatment results after IPL-PDT for NMSC [8]. However, the outcome of this treatment is difficult to compare, since different methods, treatment schedules, and evaluation criteria of clinical benefit have been used.

The use of IPL for ALA or MAL activation in PDT of NMSC has been addressed in a few studies. IPL has been mostly described as light source for the PDT of AK as part of photorejuvenation process reporting high clearance rates.

Ruiz Rodriguez et al [8] evaluated the combined use of ALA-PDT and IPL for the treatment of AKs and photodamage. Seventeen patients with a total of 38 AKs and varying degrees of photodamage underwent therapy with IPL-ALA-PDT. Two sessions with a one-month interval were performed. At three months follow-up, 87% of AKs disappeared [8]. Likewise, Avram and Goldman [9] used an IPL device for ALA-PDT to treat 17 patients with AK and photodamage resulting in a 69% decrease in AK with one IPL treatment. In both studies, photorejunative effects on the treated areas of the skin, in terms of skin texture, wrinkling, pigmentary changes, and teleangiectasias, were noted [9]. However, markedly lower rates than averages have been reported by Kim et al. in a study on the combined use of ALA-PDT with IPL for the treatment of AK exclusively [10]. Seven Korean patients with a total of 12 facial AKs underwent one session treatment with ALA-PDT using IPL. A clinical clearance rate of 50% was achieved at 12 weeks follow-up. A possible explanation for the lower AK clearance rate may be the fact that this study was performed in patients with darker skin types. Indeed, it has been reported that differences in skin pigmentation could influence PDT response, as melanin might moderate the photodynamic effect by absorbing free radicals and light [11].

The possible synergistic effect of IPL and PDT for the treatment of AK has been, also, addressed in several comparative studies. Gold et al. conducted a split face comparative study using ALA-IPL vs. IPL alone for photorejuvenation, and showed an increased clearance rate of AK with a short-contact (30 to 60 min) ALA-PDT and IPL vs. IPL alone (78% vs. 53.6%) after three sessions with a one-month interval. Additionally, improvement in several photoaging parameters was also observed [12].

A recent prospective randomized placebo-controlled study by Kohl et al. evaluated the efficacy of MAL-PDT with IPL vs. placebo-IPL for treating AK on the dorsal hands. At 10 weeks follow-up, complete AK clearance rates per hand were 54.5% after MAL-IPL and 3.0% after placebo-IPL (*p* < 0.0001) while complete AK clearance rates per lesion were 69% and 15%, respectively (*p* < 0.001). Both treatment modalities significantly improved photoaged skin of the dorsal hands and induced neocollagenesis [14].

In a previous prospective controlled study by Tadiparthi et al., MAL-IPL was also found to be more effective in treating large areas of AK compare to IPL alone; however, the AK clearance rate after MAL-IPL was marginally higher than the AK clearance rate after IPL alone (60% vs. 55%) [15].

Regarding IPL-PDT efficacy in BD and sBCC, published data are based on small case series. Hasegawa et al. treated three patients who were clinically and histopathologically diagnosed with BD. All of the patients underwent therapy with ALA-PDT using IPL. Five sessions with two-week intervals were performed. After treatment, all patients experienced transient mild edema, erythema, desquamation and crusting with healing within 10 days. They also reported pain during and after irradiation. At three-month follow-up, clinical and histopathological assessment showed complete regression of all lesions. During a one-year follow-up period no clinical signs of recurrence were observed in any of the patients [16].

Downs et al. assessed the efficacy of MAL-PDT using an IPL in 40 patients with mixed diagnoses, including AK (11 scalp lesions and 10 various), BD (9), and sBCC (10). In terms of BD and sBCC outcomes, a clearance rate of 100% was achieved at four months post-treatment. Complete clearance rate for scalp and various AKs were 91% and 100%, respectively. Only one partial recurrence in an immunocompromised patient was observed. All patients experienced warmth and mild-to-moderate pain that lasted for less than a second [17].

To date no clearly defined treatment parameters for the use of IPL in PDT are available. Haddad et al. compared various IPL light doses for ALA-PDT of AK and photodamaged skin, and demonstrated that higher IPL fluence lead to a better AK outcome, but not to an increase of improvement of photodamage [18]. However, whether higher fluences may result in higher clearance rates or increase the risk of photothermal skin damage should be further investigated.

The use of IPL as light source for PDT has become more popular for several reasons. Versatility in treating many dermatological conditions, as well as lower commercial costs and more robust technology are strong advantages for IPL devices when compared to lasers [19,20]. In addition, IPL devices have the advantage of the high skin coverage rate. Its large spot sizes allow the treatment of multiple lesions in different anatomical areas in a significantly shorter time. Further advantages to lasers and LED systems are the broadband emission of IPL devices in terms of treatment duration and the short-light pulses in terms of pain. Indeed, the shorter treatment time of IPL-PDT makes treatment more comfortable and time-efficient for both patients and medical équipe. In addition, IPL devices have been shown to be effective in the simultaneous treatment of apparent AK and perilesional sun-damaged skin [7,8]. Therefore, IPL-PDT could be an alternative therapeutic option for patients with field cancerization.

Disadvantages of IPL-PDT related to the possible inconsistency of the emitted light spectrum from pulse to pulse in particular in older IPL devices containing small capacitors bank [18]. In addition, unwanted hair reduction in the treated area can occur, given that IPL devices may be used for photoepilation.

Limitations of our study include the small sample size, the lack of comparison group, and the lack of post treatment histological assessment, as well as the short follow-up time.

## 5. Conclusions

We believe that IPL is an effective and useful alternative light source for PDT of NMSC since provides numerous advantages such as high efficacy rates, minimal discomfort, rapid treatment and recovery times, and excellent cosmetic outcomes. However, further studies are needed for developing an IPL-PDT protocol for the treatment of NMSC.

## Figures and Tables

**Table 1:** Complete clinical and dermoscopic clearance rates six months after last MAL-PDT using IPL as light source.

NMSC	Mean Pain during Treatment	Clearance Rates (%)
AK	3	18/20 (90%)
sBCC	4	4/5 (80%)
BD	4	4/4 (100%)

NMSC, nonmelanoma skin cancers; AK, actinic keratosis; sBCC, superficial BCC; BD, Bowen disease; Mean pain score on a visual 11-point scale (0, no pain; 10, insufferable pain).
